# Mapping the sialic acid-binding sites of LuIII and H-1 parvovirus

**DOI:** 10.1128/jvi.00297-25

**Published:** 2025-07-24

**Authors:** Kevin Busuttil, Nikéa Pittman, Jon Zachary, Sujata Halder, Lorena Geilen, Amriti Singh, Adam Misseldine, Paulina Kaplonek, Paul Chipman, Jaime Heimburg-Molinaro, Peter H. Seeberger, Mario Mietzsch, Antonette D. Bennett, Robert McKenna

**Affiliations:** 1 Department of Biochemistry and Molecular Biology, University of Florida 166768 , Gainesville, Florida, USA; 2 Department of Biomolecular Systems, Max-Planck Institute of Colloids and Interfaces 28321, Potsdam, Germany; 3 Institute of Chemistry and Biochemistry, Free University of Berlin 197690, Berlin, Germany; 4 Department of Surgery, Beth Israel Deaconess Medical Center, National Center for Functional Glycomics, Harvard Medical School, Center for Life Sciences 1859 , Boston, Massachusetts, USA; Cornell University Baker Institute for Animal Health, Ithaca, New York, USA

**Keywords:** protoparvovirus, glycan, receptor, oncolytic

## Abstract

Oncolytic protoparvoviruses, including LuIII, H-1 parvovirus (H-1PV), and the prototypic strain of minute virus of mice (MVMp), can target and destroy cancer cells. Host cell targeting is based largely on the identification and interaction of the virus with the primary receptor. Previously, it has been shown that MVMp and H-1PV bind to sialic acid (SIA), which is the primary glycan receptor. This study investigates whether LuIII also utilizes a similar glycan for host cell attachment. Microarray analysis confirmed that α2-3-linked SIA is a shared receptor requirement for cell binding for all three viruses. Three glycans were identified in the array, namely, Neu5Acα2-3Galβ1-4GlcNAcβ1-3Galβ1-4GlcNAc, Neu5Acα2-3Galβ1-4GlcNAcβ1-3Galβ1-4GlcNAcβ1-3Galβ1-4GlcNAc, and Neu5Acα2-3Galβ1-4(Fucα1-3)-GlcNAcβ1-3-Galβ1-4(Fucα1-3)-GlcNAc. The cryo-EM structures of the LuIII and H-1PV glycan complexes were determined to resolutions ranging from 2.57 to 2.88 Å. Small structural perturbations were observed between the cryo-EM map and the previous X-ray crystallographic maps for H-1PV, including several histidine residues within the HI loop. Overall, LuIII and H-1PV utilize a shared SIA recognition pocket near the icosahedral twofold axis adjacent to (but not overlapping with) the known MVMp SIA binding site. In addition, structural differences between the major capsid protein (VP2) of LuIII, H-1PV, and MVMp all clustered around these glycan-binding pockets. This structural phenotype may contribute to the differences observed in tumor cell killing efficiency among the rodent protoparvoviruses.

**IMPORTANCE:**

Oncolytic viruses could provide a safe alternative for targeting aggressive tumors that evade standard therapies. While rodent protoparvoviruses are innocuous in non-cancerous cells, they carry out efficient cell killing in tumors. Differences in cell tropism and killing efficiency are determined by the viral capsid proteins; thus, structural studies provide insight into understanding the protoparvovirus infection in both wild-type and cancerous cells. Binding to extracellular sialic acid (SIA) initiates cell entry for protoparvoviruses H-1PV, MVMp, and this is also hypothesized for LuIII. This study investigates the structures of LuIII and H-1PV in the presence of their glycan receptors to identify and map the capsid loci that are responsible for this interaction. This knowledge may aid future capsid engineering to improve oncolytic targeting efficiency.

## INTRODUCTION

The *Parvoviridae* are a family of small, single-stranded DNA viruses that package a linear genome into a non-enveloped T = 1 icosahedral capsid (1). Within the *Protoparvovirus* genus, a subgroup of viruses has inherent oncolytic activity against human tumors, despite mice and other rodents being their natural hosts. These viruses include the rodent protoparvoviruses LuIII, H-1 parvovirus (H-1PV), and the prototypic strain of minute virus of mice (MVMp), all of which are not pathogenic to humans (2). These viruses demonstrate autonomous replication but are S-phase dependent, due to a need for host co-factors to enable viral DNA synthesis, transcription, and/or translation (3). In addition, several proteins that are upregulated in cancer cells have also been shown to aid in the control of virus gene expression (4). For these reasons, oncolytic protoparvoviruses preferentially infect transformed or tumor-derived cells, leading to apoptosis and virus-mediated activation of anti-tumor immunity (5, 6). Expectedly, many of the rodent protoparvoviruses were isolated from transplantable tumors during their initial discovery (7–10). Of these rodent viruses, H-1PV, MVMp, and, more recently, LuIII are the most widely studied oncolytic protoparvoviruses, although only H-1PV has progressed to clinical studies (2). The oncolytic ability of rodent protoparvoviruses is dependent on the type of cancer and the cell line derived from the cancer (2, 6). The non-structural 1 (NS1) protein controls viral replication in the tumors, cytotoxicity, and viral fitness (6, 11). Notably, differences in tumor cell killing are observed upon exchanging the viral protein 2 (VP2) among H-1PV, LuIII, MVMp, and others to create chimeric viruses (2, 12, 13). This suggests that the VP2 of these protoparvoviruses contributes to tumor cell death; however, the amino acids responsible for this phenotype are unknown.

The protoparvoviruses package a 5.2 kb genome which encodes three non-structural (NS1, NS2, and the small alternatively translated [SAT]) proteins, and two VPs (VP1 and VP2) (1). Sixty copies of VP1 and VP2 self-assemble a T = 1 icosahedral capsid in a ~ 1:10 ratio. The VP2 of genome-containing particles is cleaved at its N-terminus to produce VP3, yielding a capsid with a ~1:1:10 ratio of VP1:VP2:VP3 (14). The overlapping VP sequences share the C-terminus, with N-termini of variable length. The first 142 residues of the N-terminus of VP1 are unique to VP1 (VP1u) and contain a phospholipase A2 (PLA_2_) domain, which is important for infectivity (15). However, to date, structural studies have only visualized VP2 (starting with residue number ~38 among the protoparvoviruses) (16). The remaining VP1/VP2 N-termini are unresolved, most likely because these regions are flexible, inherently disordered, or have multiple conformations that do not conform to the enforced icosahedral averaging during the structure determination. The capsid structures of MVMp, H-1PV, and LuIII have been previously determined, and they share features common among the animal protoparvoviruses (16). These include channels at the icosahedral fivefold axes, protrusions at each threefold axis, depressions at the twofold axes, and canyons surrounding the fivefold channels. In addition, a region termed the 2/5 wall separates the twofold depressions from their neighboring fivefold canyons (16). Structural comparison of VP2 among the *Parvoviridae* demonstrates that the core capsid is conserved (including an α-helix and an eight β-stranded, anti-parallel jelly roll motif). However, regions of sequence and structural diversity cluster on the capsid surface within previously defined variable regions (VRs) that connect the jelly roll motif and create the surface topology. These surface features are both antibody- and receptor-binding sites and have been shown to control virus tropism (16–18).

The MVMp and H-1PV capsids initiate cell binding through recognition of a receptor possessing a negatively charged terminal SIA sugar motif (16, 19). Although several protoparvoviruses bind SIA, the specific capsid residues involved are unknown for many of these viruses. This includes H-1PV and several non-oncolytic viruses such as porcine parvovirus (PPV), canine parvovirus (CPV), and feline panleukopenia virus (FPV) (17, 19–21). In fact, to date, only the MVM and CPV receptor-binding site has been fully mapped and confirmed by mutagenesis (16, 18, 22). Both MVMp and the immunosuppressive (MVMi) strains recognize α2-3-linked SIA, specifically the N-acetyl neuraminic acid (Neu5Ac) commonly found on human cells (23). Although the glycoprotein receptor for MVMp is unknown, H-1PV cell recognition and oncolysis were recently linked to laminin γ1 in an SIA-dependent manner (24). Furthermore, SIA binds on the surface of MVMp within a cavity at the twofold depression (19). Interestingly, many of the VP2 residues that control MVM differences in virulence, cell tropism, or host range also map to the SIA binding pocket (18, 23). Substitutions of the homologous residues in H-1PV revealed that cell binding is controlled by at least two amino acids in MVMp (H374/K368 and I368/I362) for H-1PV/MVMp, respectively (19). However, glycan receptor recognition has not yet been characterized for LuIII, and the exact capsid loci involved in H-1PV SIA interactions remain unknown.

Glycan receptors displayed on cell surfaces represent diverse combinations of carbohydrates (glycans) connected via α- or β-glycosidic linkages. Additional complexity arises from the modification of functional groups within each glycan, such as the N-acetyl, hydroxyl, or glycerol groups on SIA (25, 26). Glycan expression profiles vary by species, cell type, and changes during development and disease, such as cancer (27). Thus, the goal of this study is to provide insights into *Protoparvovirus* cell tropism by defining specific glycan structures utilized for cell binding and identifying the important residues involved. LuIII, H-1PV, and MVMp similarly hemagglutinate erythrocytes and often infect the same cell types (2). In addition, many surface residues within MVMp’s SIA-binding pocket are conserved, leading to the hypothesis that all three viruses might utilize the same or similar receptor.

Virus-like particles (VLPs) composed of VP2 (that are void of DNA) are useful for probing capsid-receptor interactions and are indistinguishable from wild-type capsids by biochemical, antigenic, or structural analysis (16, 28). Therefore, VLPs for LuIII, H-1PV, and MVMp were tested by glycan microarray screening and *in vitro* cell-binding experiments. From these results and through comparison with previous studies of MVMp, all three viruses were shown to recognize Neu5Acα2-3Galβ1-4GlcNAc, which is abbreviated s(LN), which is upregulated in tumor cells within the Sialyl Lewis^x^ (s(Le^x^)) motif. To resolve unanswered questions of H-1PV and LuIII glycan recognition, VLPs of both viruses in complex with Neu5Acα2-3Galβ1-4GlcNAcβ1-3Galβ1-4GlcNAcβ1-3Galβ1-4GlcNAc, which is s(LN)_3_, or Neu5Acα2-3Galβ1-4(Fucα1-3)-GlcNAcβ1-3-Galβ1-4(Fucα1-3)GlcNAc, which is s(Le^x^)_2_, were analyzed by cryo-electron microscopy (cryo-EM). The structures illustrated that both LuIII and H-1PV share a glycan receptor-binding site different from MVM. This SIA-binding site is surrounded by structurally diverse VRs and is located proximally to the twofold icosahedral axis bordering the already mapped MVM receptor-binding region.

## RESULTS

### Characterization of LuIII and H-1PV glycan recognition by microarray screening

**Fig 1:**
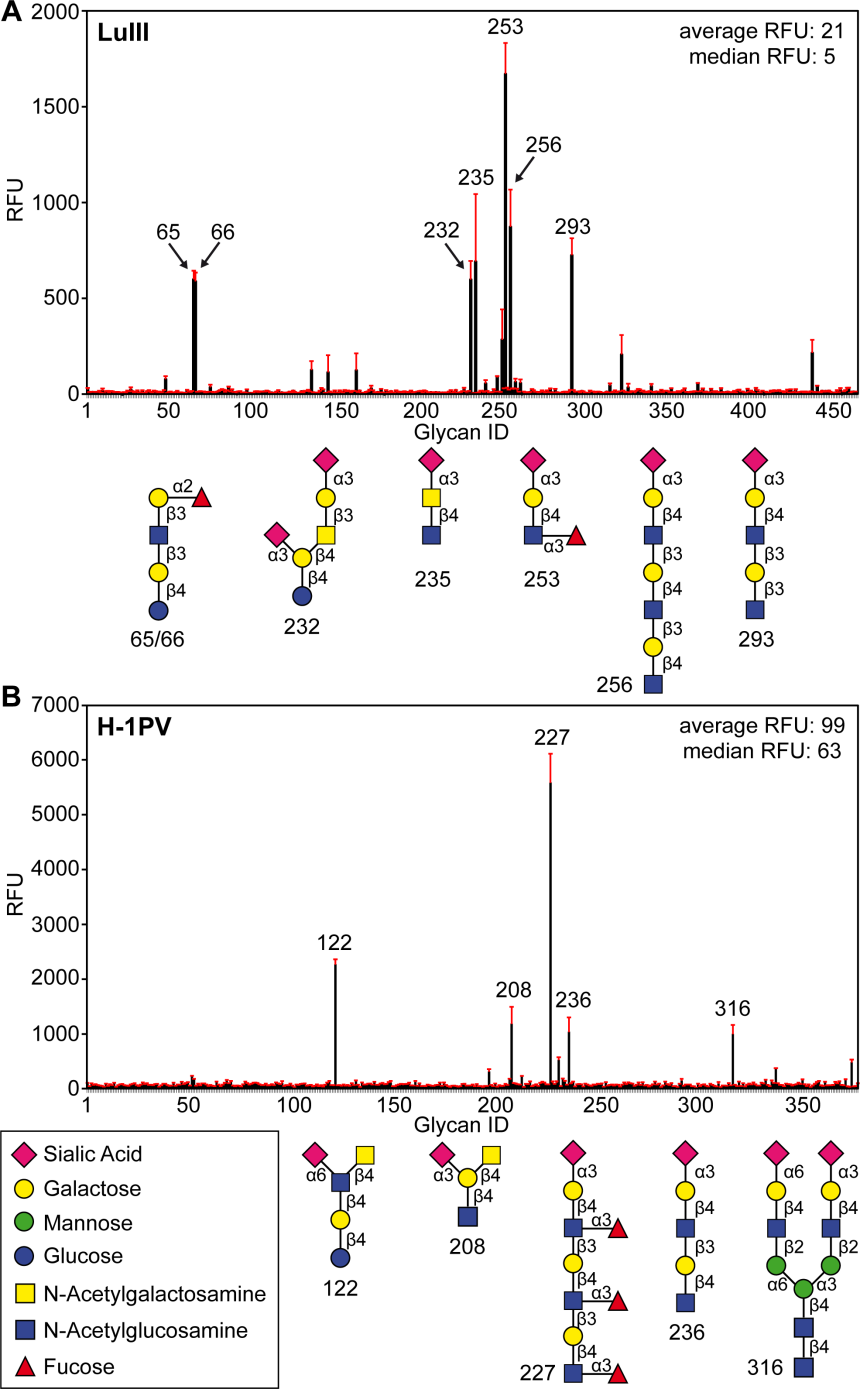
CFG glycan microarray screening for LuIII and H-1PV. (**A**) Results from the LuIII glycan screen with seven significant glycan structures depicted in cartoon representation along the x-axis. (**B**) Results from the H-1PV glycan screen with five significant glycan structures depicted in cartoon representation along the x-axis. Peaks (in black) indicate the average relative fluorescent signal for binding to specified glycans on the x-axis, with standard deviations in red. Each screening was done independently.

Microarray screening of LuIII capsids using the Consortium for Functional Glycomics (CFG) array (Version 4.1) identified six glycans of interest out of the 465 glycans screened (Fig. 1A; Table 1). The readouts of the six glycans ranged between 600 and 1750 RFU, which is consistent with previous glycan array data obtained for other parvoviruses and their appropriate glycan receptors (16). LuIII preferentially bound α2-3-linked SIA (3′SIA) within linear epitopes containing galactose (Gal)/N-acetyl galactosamine (GalNAc), and N-acetyl glucosamine (GlcNAc) (Fig. 1A; Table 1). Accordingly, LuIII bound to s(LN)_2_ (Glycan 293) and s(LN)_3_ (Glycan 256). Neu5Acα2-3Galβ1-4(Fucα1-3) GlcNAc (Glycan 253 or s(Le^x^)) produced the highest signal on the LuIII glycan array. The s(Le^x^) motif represents a fucosyl (Fuc) modification of s(LN) and is upregulated in cancer cells (26). LuIII also recognized other sialylated motifs that deviate from the s(LN) trisaccharide core by the addition of N-acetyl galactosamine (GalNAc) or glucose (Glc). This included Neu5Acα2-3(Neu5Acα2-3Galβ1-3GlcNAcβ1-4)Galβ1-4Glc (Glycan 232) and Neu5Acα2-3GalNAcβ1-4GlcNAc (Glycan 235). In addition, two non-sialylated glycans (Glycan 65 and 66, differing only in their flexible linker region) also bound to LuIII using the Fucα1-2Galβ1-3GalNAc motif. LuIII did not recognize any glycans that terminated in either α2-6-linked SIA (6′SIA), other sugars (such as GlcNAc, GalNAc, Glc, or mannose (Man)), or polysialylated Neu5Acα2-8Neu5Ac motifs. Furthermore, sulfation of Gal and GlcNAc functional groups inhibited LuIII glycan recognition, despite the presence of terminal 3′SIA within these sugars; therefore, LuIII appears to preferentially bind to 3′ terminal SIA.

**TABLE 1:** CFG glycan array data for LuIII and H-1PV^*a*^

VLP	ID	Glycan structure	RFU	STDEV	SEM	% CV
LuIII	253	Neu5Acα2-3Galβ1-4(Fucα1-3)GlcNAcβ-Sp8/(s(Le^x^)-)	1,685	423	212	25
	256	Neu5Acα2-3Galβ1-4GlcNAcβ1-3Galβ1-4GlcNAcβ1-3Galβ1-4GlcNAcβ-Sp0/(s(LN)_3_-)	878	393	197	45
	293	Neu5Acα2-3Galβ1-4GlcNAcβ1-3Galβ1-3GlcNAcβ-Sp0/(s(LN)_2_-)	728	180	90	25
	235	Neu5Acα2-3GalNAcβ1-4GlcNAcβ-Sp0	695	713	356	103
	65	Fucα1-2Galβ1-3GlcNAcβ1-3Galβ1-4Glcβ-Sp8	601	93	46	15
	232	Neu5Acα2-3(Neu5Acα2-3Galβ1-3GalNAcβ1-4)Galβ1-4Glcβ-Sp0	600	197	98	33
	66	Fucα1-2Galβ1-3GlcNAcβ1-3Galβ1-4Glcβ-Sp10	590	93	47	16
H-1PV	227	Neu5Acα2-3Galβ1-4(Fucα1-3)GlcNAcβ1-3Galβ1-4(Fucα1-3)GlcNAcβ1-3Galβ1-4(Fucα1-3)GlcNAcβ–Sp0/(s(Le^x^)_3_-)	5,574	1,066	533	19
	122	Galβ1-3(Neu5Acα2-6)GlcNAcβ1-4Galβ1-4Glcβ-Sp10	2,261	193	97	9
	208	Neu5Acα2-3(GalNAcβ1-4)Galβ1-4GlcNAcβ-Sp8	1,178	629	314	53
	236	Neu5Acα2-3Galβ1-4GlcNAcβ1-3Galβ1-4GlcNAcβ-Sp0/(s(LN)_2_-)	1,028	541	270	53
	316	Neu5Acα2-3Galβ1-4GlcNAcβ1-2Manα1-3(Neu5Acα2-6Galβ1-4GlcNAcβ1-2Manα1-6)Manβ1-4GlcNAcβ1-4GlcNAcβ-Sp12	991	338	169	34

^*a*^ Neu5Ac, sialic acid; Gal, galactose; Fuc, fucose; GalNAc, N-acetyl-d-galactosamine; GlcNAc, N-acetyl-d-glucosamine; Glc, glucose; Man, mannose; SP0, -CH₂CH₂NH₂; SP8, -CH₂CH₂CH₂NH₂; Sp10, -NHCOCH₂NH; SP12, asparagine.

In a separate experiment, H-1PV was screened using a different version of the CFG array (Version 3.1), identifying 5 out of 377 glycans as binding targets (Fig. 1B; Table 1). Similar to LuIII, s(Le^x^) and s(LN) motifs were recognized as the H-1PV glycan-binding partners, with s(Le^x^)_3_ (Glycan 227) showing the strongest signal (Fig. 1B; Table 1). H-1PV interacted with s(LN)_2_ (Glycan 236), three additional terminally sialylated glycans: Galβ1-3(NeuAcα2-6)GlcNAcβ1-4Galβ1-4Glc (Glycan 122), NeuAcα2-3(GalNAcβ1-4)Galβ1-4GlcNAc (Glycan 208), and a biantennary glycan, Neu5Acα2-3Galβ1-4GlcNAcβ1-2Manα1-3(Neu5Acα2-6Galβ1-4GlcNAcβ1-2Manα1-6)Manβ1-4GlcNAcβ1-4GlcNAcβ (Glycan 316). Each glycan recognized by H-1PV contained at least one 3′SIA, except for 6′SIA in Glycan 122. In addition, H-1PV did not bind to polysialylated carbohydrate chains or those terminating in Gal, Fuc, GlcNAc, GalNAc, Man, or Glc.

### LuIII, H-1PV, and MVM utilize a shared sialic acid receptor

**Fig 2:**
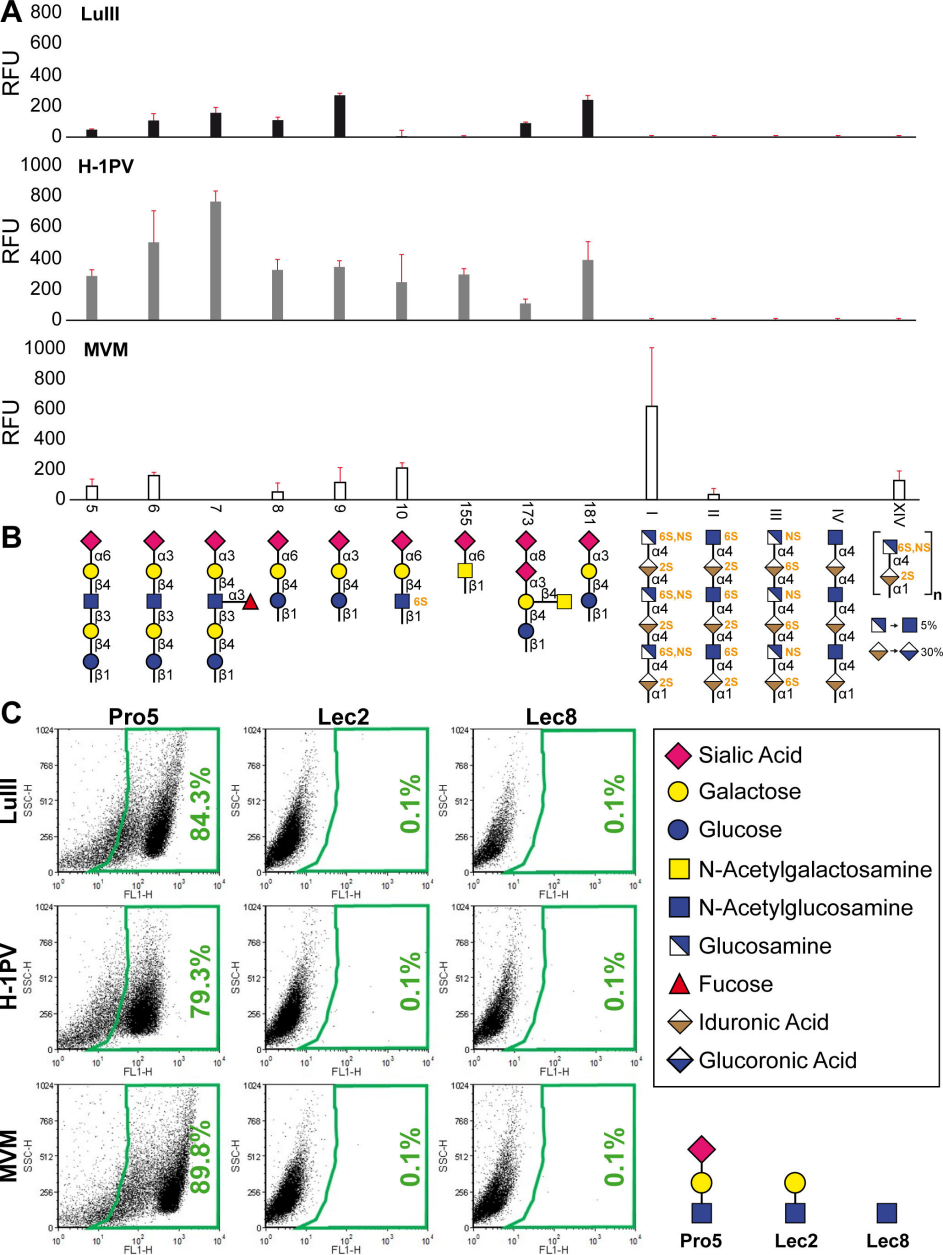
Comparison of LuIII, H-1PV, and MVM glycan recognition. (**A**) Results from microarray screening utilizing the Max-Planck Institute glycan library containing 23 glycans, including 4 synthetic heparins (I, II, III, and VIII) and a low-molecular-weight natural heparin (XIV). (**B**) The corresponding glycans are depicted in cartoon representation. (**C**) LuIII, H-1PV, and MVM preferentially bind terminal SIA on Pro-5 cells, compared to Lec-2 or Lec-8. A representative experiment is shown (*n* = 3).

To enable direct comparison of binding preferences, all three viruses were screened on the same glycan array, with 23 glycans in total, including seven unbranched sugars that displayed terminal SIA (Fig. 2A and B; Table 2). LuIII and H-1PV recognized all the terminally 3′-sialylated glycans, while MVM only recognized two out of the four. Only LuIII and H-1PV recognized Neu5Acα2-3Galβ1-4(Fucα1-3)GlcNAcβ1-3Galβ1-4Glc (Glycan 7), which contains the s(Le^x^) motif. Differing from the CFG glycan array, all of the tested viruses recognized multiple sugars terminating in 6′SIA (such as Glycans 5, 8, 10, or 155), and LuIII and H-1PV both bind to the polysialylated Neu5Acα2-8Neu5Acα2-3(GalNAcβ1-4)Galβ1-4Glc motif known as GD2 (Glycan 173). In addition, this glycan library included synthetic (I-VIII) or natural heparins (XIV). While LuIII and H-1-PV did not recognize any heparins, binding of MVM to heparins I and XIV was observed (Fig. 2A and B; Table 2). For H-1PV, interaction with iduronic acid monosaccharides was also detected.

**TABLE 2:** Max-Planck Institute glycan array data for MVM, LuIII, and H-1PV^*a*^

VLP	ID	Glycan structure	RFU	STDEV	SEM	% CV
LuIII	9	Neu5Acα2-3Gαlβ1-4Glcβ-AH	267	44	15	16
	181	Neu5Acα2-3Gαlβ1-4Glcβ-AP	237	14	5	6
	7	Fucα1-3(Neu5Acα2-3Gαlβ1-4)GlcNAcβ1-3Gαlβ1-4Glcβ-AH	151	23	8	15
	6	Neu5Acα2-3Gαlβ1-4GlcNAcβ1-3Gαlβ1-4Glcβ-AH	106	37	12	35
	8	Neu5Acα2-6Gαlβ1-4Glcβ-AH	105	13	4	12
	173	Neu5Acα2-8Neu5Acα2-3(GαlNAcβ1-4)Gαlβ1-4Glcβ-AP	88	26	9	29
	5	Neu5Acα2-6Gαlβ1-4GlcNAcβ1-3Gαlβ1-4Glcβ-AH	43	37	12	87
H-1PV	7	Fucα1-3(Neu5Acα2-3Gαlβ1-4)GlcNAcβ1-3Gαlβ1-4Glcβ-AH	755	73	24	10
	6	Neu5Acα2-3Gαlβ1-4GlcNAcβ1-3Gαlβ1-4Glcβ-AH	494	205	68	41
	181	Neu5Acα2-3Gαlβ1-4Glcβ-AP	382	121	40	32
	9	Neu5Acα2-3Gαlβ1-4Glcβ-AH	333	48	16	15
	8	Neu5Acα2-6Gαlβ1-4Glcβ-AH	316	71	24	23
	155	Neu5Acα1-6GαlNAcα-AP	283	44	15	15
	5	Neu5Acα2-6Gαlβ1-4GlcNAcβ1-3Gαlβ1-4Glcβ-AH	278	42	14	15
	10	Neu5Acα2-6Gαlβ1-4GlcNAc-6-sulfαteβ-AH	235	185	62	79
	173	Neu5Acα2-8Neu5Acα2-3(GαlNAcβ1-4)Gαlβ1-4Glcβ-AP	99	35	12	35
MVM	I	GlcNS6Sα1-4IdoA2Sα1-4GlcNS6Sα1-4IdoA2Sα1-4GlcNS6Sα1-4IdoA2Sα1-4GlcNS6Sα1-4IdoA2Sα-AS	608	937	312	154
	10	Neu5Acα2-6Gαlβ1-4GlcNAc-6-sulfαteβ-AH	203	36	12	18
	6	Neu5Acα2-3Gαlβ1-4GlcNAcβ1-3Gαlβ1-4Glcβ-AH	153	24	8	16
	XIV	(GlcNS6Sα1-4IdoA2Sα1)ₙ-AH	124	63	21	51
	9	Neu5Acα2-3Gαlβ1-4Glcβ-AH	109	102	34	93
	5	Neu5Acα2-6Gαlβ1-4GlcNAcβ1-3Gαlβ1-4Glcβ-AH	84	49	16	58
	8	Neu5Acα2-6Gαlβ1-4Glcβ-AH	47	61	20	131
	II	GlcNAc6Sα1-4IdoA2Sα1-4GlcNAc6Sα1-4IdoA2Sα1-4GlcNAc6Sα1-4IdoA2Sα-AP	32	43	14	135

^*a*^ Neu5Ac, sialic acid; Gal, galactose; Fuc, fucose; GalNAc, N-acetyl-d-galactosamine; GlcNAc, N-acetyl-d-glucosamine; GlcN, glucosamine; Glc, glucose; Man, mannose; Ido, iduronic acid; AP, -CH₂CH₂CH₂CH₂CH₂NH₂; AH, -CH₂CH₂CH₂CH₂CH₂CH₂NH₂; AS, -CH₂CH₂CH₂CH₂CH₂SO₂CH₂CH₂NH₂.

Glycan microarrays are useful for screening multiple potential binding partners at once, but array surfaces do not mimic the biological context of the cell. Therefore, to better understand the role of SIA during cell binding, fluorescence-labeled LuIII, H-1PV, and MVM VLPs were incubated with Chinese hamster ovary (CHO) cell lines that display differential glycan profiles. Binding was detected by flow cytometry after viral adsorption to Pro-5, Lec-2, or Lec-8 cell surfaces. It was shown that LuIII, H-1PV, and MVMp bound specifically to Pro-5 cells, which express terminal SIA. Alternatively, Lec-2 and Lec-8 cell lines display terminal Gal or GlcNAc, respectively, and did not show any binding by the labeled capsids (Fig. 2C).

### Sialic acid binds to the wall of the twofold icosahedral axis on the surface of LuIII

**Fig 3:**
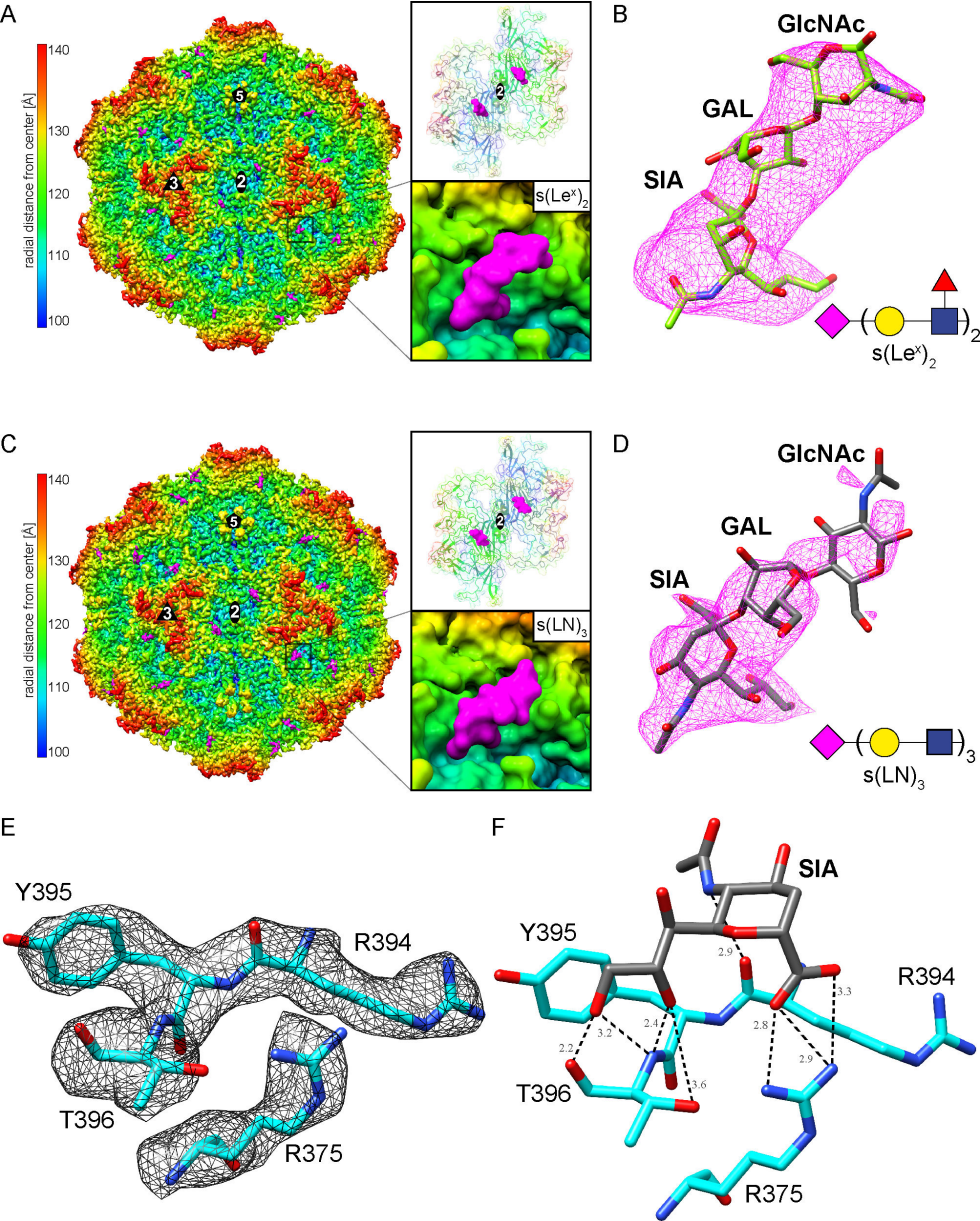
s(LN)_3_ binding site of LuIII. (**A**) Capsid structure of LuIII with s(Le^x^)_2_ densities in magenta. The capsid surface is viewed down the icosahedral twofold axis and is colored according to radial distance from the particle center (blue to red), as indicated by the scale bar. Boxes to the right highlight the glycan’s binding position relative to the twofold depression. (**B**) The modeled s(Le^x^)_2_ fit into its density map contoured at a sigma (σ) threshold level of 0.2 for maximum visibility. Due to the lack of density for the branching fucose or subsequent Gal-(Fuc)-GlcNAc residues, these sugars were left unmodeled. (**C**) Capsid structure of LuIII with s(LN)_3_ densities in magenta. The capsid surface is viewed down the icosahedral twofold axis and is colored according to the radial distance from the particle center (blue to red), as indicated by the scale bar. Boxes to the right highlight the glycan’s binding position relative to the twofold depression. (**D**) The modeled s(LN)_3_ fit into its density map contoured at a sigma (σ) threshold level of 0.7 for maximum visibility. Due to the lack of density for the distal Gal-GlcNAc-Gal-GlcNAc residues, these sugars were left unmodeled. (**E**) The modeled LuIII residues of the binding pocket are shown inside their density map at a sigma (σ) threshold level of 3.5. (**F**) The terminal SIA (gray) from s(LN)_3_ shown with distances from LuIII residues (tan). Both are shown as stick representations and colored according to atom type: C, gray or tan; O, red; N, blue. All distances are shown in angstroms. Images were generated using Chimera.

To further investigate the receptorbinding interactions of LuIII and its glycan-binding partners, LuIII VLPs were complexed with s(LN)_3_ or s(Le^x^)_2_, and cryo-EM data were collected. While mono-s(Le^x^) (Glycan 253) showed the highest binding signal in the glycan array, we selected the related glycan s(Le^x^)_2_ for structural studies under the assumption that it would similarly engage with the capsid due to the presence of the same terminal motifs as well as its potential to provide additional interaction sites with the capsid, possibly enhancing the stability of the complex for cryo-EM visualization. High-resolution structures of LuIII-s(LN)_3_ and LuIII-s(Le^x^)_2_ complexes were determined from 56,731 and 38,079 capsids, to 2.66 Å and 2.88 Å resolution, respectively (Table 3). Comparative analysis of the LuIII-s(Le^x^)_2_ and LuIII-s(LN)_3_ structures with the EM map of LuIII (PDB ID: 6B9Q) revealed additional density located on the capsid surface within a shallow cavity at the base of the threefold protrusion, surrounded by the twofold depression and 2/5 wall of the capsid. The additional densities of the LuIII-s(LN)_3_ and LuIII-s(Le^x^)_2_ complexes were fitted with the models of their respective ligands. The fit of the model in the map illustrated as the map-to-model cross-correlation (CC) values were 0.89 and 0.87, respectively (Table 3). The ligand density of LuIII-s(LN)_₃_ complex provided more detail at higher sigma levels compared to the LuIII-s(Le^x^)_2_ complex. Given their equivalent binding modes, this suggests that both glycans interact with the same amino acids. Subsequently, all structural analyses were focused on the LuIII-s(LN)₃ complex (Fig. 3A through D).

**TABLE 3:** Cryo-EM structure determination and refinement statistics

Parameter	LuIII-s(LN)_3_	LuIII-s(Le^x^)_2_	H-1PV	H-1PV-s(Le^x^)_2_
Data collection and processing				
Total no. of micrographs	403	876	546	838
Defocus range (µm)	1.0–3.0	0.90–3.1	0.81–2.9	1.1–2.2
Electron dose (e-/Å^2^)	75	75	75	75
No. of frames/micrograph	50	50	50	50
Pixel size (Å^2^/pixel)	1.05	1.05	1.05	1.05
No. of particles used for final map	38,079	56,731	32,893	30,120
Inverse B-factor used for final map (Å^2^)	20	20	50	50
Resolution of final map (Å)	2.66	2.88	2.59	2.57
Residue range	37–587	37–587	35–593	35–593
RMSD				
Bond distances (Å)	0.01	0.01	0.01	0.01
Bond angles (°)	0.835	1.03	0.866	0.927
Map CC	0.887	0.87	0.701	0.743
All atom clash score	9.59	9.74	8.63	7.62
Ramachandran plot				
Favored (%)	98.2	97.8	98.4	98.4
Allowed (%)	1.8	2.0	1.6	1.6
Outliers (%)	0	0.2	0	0
Rotamer outliers (%)	0.2	0.6	0.2	0.2
No. of C-beta deviations	0	0	0	0

Proximal to the LuIII capsid surface, the density corresponding to 3′SIA of s(LN)_3_ was well-ordered, becoming visible at a contour level of 2.0σ. By contrast, the density for the subsequent glycan chain was observed to get increasingly weaker with increased radial distance from the capsid (Fig. 3D). Although densities corresponding to the Gal and GlcNAc residues following the sialic acid became visible at contour levels of 1.5σ and 1.0σ, respectively, no discernible density was detected for the distal Gal-GlcNAc-Gal-GlcNAc glycan chain, and therefore, these sugars were left unmodeled. The LuIII capsid in the complex map is well-ordered and facilitates the reliable placement of the amino acid side chains into the density during model building (Fig. 3E). Comparison of the LuIII (PDB ID: 6B9Q) structure and the LuIII complex structures did not reveal any significant differences except for the additional glycan density. Structural superposition of the bound and unbound structures had overall Cα root mean squared deviation (RMSD) values of 0.35 Å. Minor changes were observed for the main chain (aas 158–164, VRII) and for several side chain conformations (aas 383–384, 476, and 517), which were unrelated to the glycan-binding region.

The interactions of SIA with the LuIII capsid are predicted to include a salt bridge between the C1 carboxylate and R375, and several hydrogen bonds to R394 and T396 (Fig. 3F; Table 4). No interactions between the capsid and Gal or GlcNAc were detected, likely explaining the increasing disorder observed in the density map. Calculations with PDBePISA revealed that 13 residues were partially or completely buried by the ligand, which are located primarily within a single VP monomer (R375, Y378, D393, R394, Y395, T396, D398, R563, V575, and P576), with minor contributions from a neighboring threefold-related VP (Q320, G321, and N323) (Fig. 5). Overall, the total buried surface area (BSA) between the two VP chains and SIA is predicted to be ~200 Å^2^, with Gal and GlcNAc contributing another ~20 Å^2^.

**TABLE 4:** VP2 residues involved in glycan recognition for LuIII and H-1PV^*b*^

LuIII			H-1PV			MVM
Residue	Distance (Å)	BSA (Å^2^)	Residue	Distance (Å)	BSA (Å^2^)	Residue
R375^*a*^	2.8, 2.9, 3.3	24.2, 9.2_GAL_	R381^*a*^	2.6, 2.6, 3.8_GAL_	19.7, 24.3_GAL_	R375
D393	NID^*c*^	27.5	E400	3.7	39.9	E394
R394	2.9	30.7, 0.1_GAL_	R401	2.7	27.1, 2.2_GAL_	R395
T396	2.2, 2.4, 3.2	21.6	T403	3.3	17.5	T397

^*a*^ Residues are involved in salt bridges.

^*b*^ All interactions and buried surface area (BSA) were calculated by PDBePISA. Distances are given for predicted hydrogen bonds. Rows are organized via structural alignment.

^*c*^ NID, no interaction detected.

### H-1PV and LuIII utilize the same pocket for sialic acid recognition

Previously, the capsid structure of H-1PV was determined by X-ray crystallography (29). To identify the glycan-binding sites on its capsid, VLPs were complexed with s(Le^x^)_2_, and cryo-EM data were collected. While s(Le^x^)_3_ (Glycan 227) showed the highest binding signal in the glycan array, we selected the related glycan s(Le^x^)_2_ for structural studies under the assumption that it would similarly engage with the capsid due to the presence of identical terminal motifs. For direct comparison, cryo-EM data on VLPs in the absence of any glycan were also collected. Utilizing 30,120 and 32,893 individual capsid images, the H-1PV capsids with and without s(Le^x^)_2_ were reconstructed to 2.57 Å and 2.59 Å resolutions, respectively (Table 3). Both structures exhibited well-ordered density for the capsid, facilitating the reliable building of the VP models. For H-1PV in the absence of s(Le^x^)_2_, the overall Cα RMSD of the VP was 0.44 Å when superposed onto the previously determined H-1PV crystal structure (PDB ID: 4G0R). As a comparison, superposition of the cryo-EM-derived structures with and without s(Le^x^)_2_ resulted in a Cα-RMSD of 0.28 Å with no structural differences observed. However, between the X-ray and cryo-EM structures, several conformational differences were visible (Fig. S1). The apex of the DE loop showed an alternative conformation with a significant shift of up to 4.5 Å (Fig. S1). This is likely related to the fact that in the X-ray structure, residues 166–167 were disordered and would not allow for the fitting of the VP model. By contrast, this density in the cryo-EM map was better ordered and permitted the reliable building of the complete DE loop. Similarly, three additional amino acids at the N-terminus were built, which were not observed in the crystallographic H-1PV structure (29). Furthermore, several residues demonstrated alternate side chain conformations within the cryo-EM structure. This was observed for a stretch of residues within the HI loop (518–524), exhibiting a local RMSD value up to 1.06 Å (Fig. S1). Several histidine residues also exhibited additional density extending from the imidazole ring (Fig. S1). This is the case in approximately half of all histidine residues within H-1PV: 65, 86, 93, 348, 448, 483, or 488 (visible at 2-4σ) and 579 (1-2σ). These are positioned either at the capsid interior or exterior, primarily clustering around the 3-fold axis or along the 2/5-fold wall (H579). The same observations were made for residues fitted to the H-1PV-s(Le^x^)_2_ map. LuIII also displays additional densities at some of its homologous histidine residues, including 52, 72, 87, 137, 141, 342, 368, 457, and 476. Interestingly, these densities were visible at a contour level of 2-4σ in the LuIII-s(LN)_3_ map, while only some were visible at a level of 1-2σ in the LuIII-s(Le^x^)_2_ map, likely due to the lower resolution.

**Fig 4:**
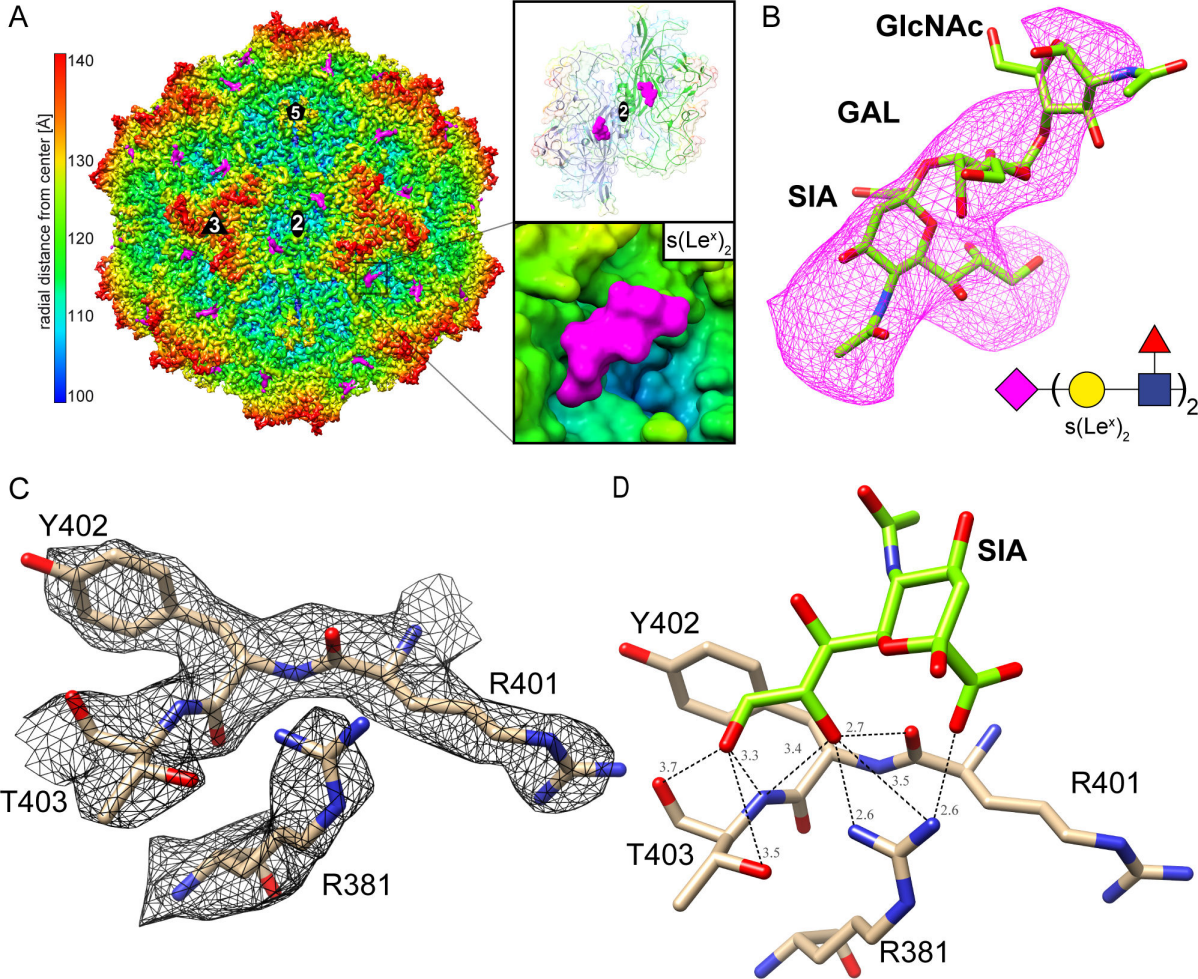
s(Le^x^)_2_ Binding site of H-1PV. (**A**) Capsid structure of H-1PV with s(Le^x^)_2_ densities in magenta. The capsid surface is viewed down the icosahedral twofold axis and is colored according to radial distance from the particle center (blue to red), as indicated by the scale bar. Boxes to the right highlight the glycan’s binding position relative to the twofold depression. (**B**) The modeled s(Le^x^)_2_ fit into its density map contoured at a sigma (σ) threshold level of 0.6 for maximum visibility. Due to the lack of density for the branching fucose or subsequent Gal-(Fuc)-GlcNAc residues, these sugars were left unmodeled. (**C**) The modeled H-1PV residues of the binding pocket are shown inside their density map at a sigma (σ) threshold level of 3.5. (**D**) The terminal SIA (green) from s(Le^x^)_2_ shown with distances from H-1PV residues (tan). Both are shown as stick representations and colored according to atom type: C, green or tan; O, red; N, blue. All distances are shown in angstroms. Images were generated using Chimera.

**Fig 5:**
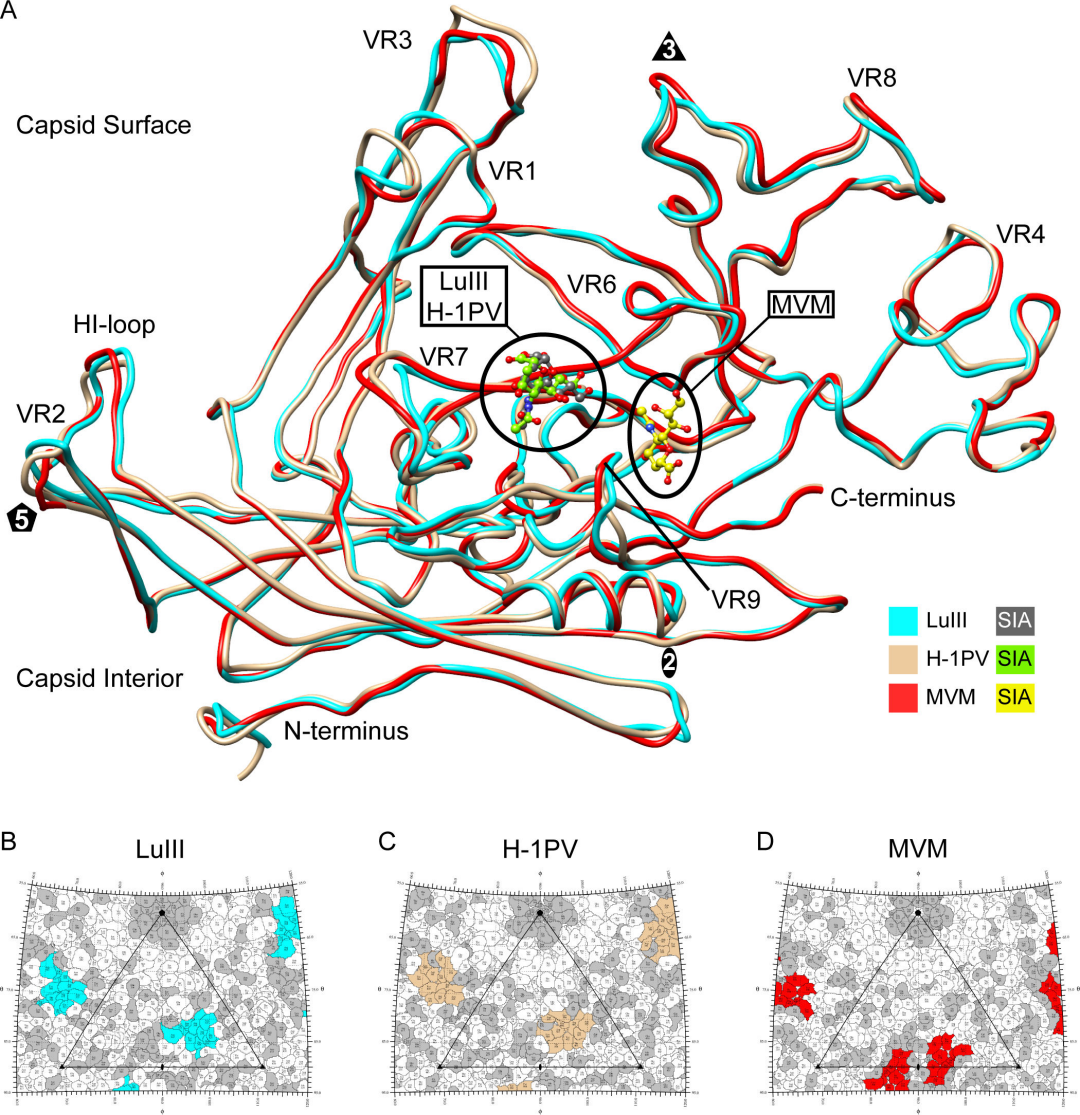
Comparison of SIA-binding sites. (**A**) Structural superposition of VP2 monomers from LuIII, H-1PV, and MVM with their individual SIA modeled as ball and sticks. The colors of each are as indicated by the legend. (**B–D**) 2D projection roadmaps of the exterior surfaces of LuIII, H-1PV, and MVM, with their buried surface areas (BSA) highlighted in cyan, tan, or red, respectively. The icosahedral axes are identified along the perimeter of the viral asymmetric unit (large triangle) by either an elliptical (twofold), a pentagon (fivefold), or a triangle (threefold). Gray highlights indicate positions of no conservation between the three viruses. Images were generated in Chimera and RIVEM.

In the H-1PV-s(Le^x^)_2_ map, additional densities not attributed to the capsid (or histidines) were visible near the twofold depression in the same capsid location as s(LN)_3_ and s(Le^x^)_2_ in the LuIII capsid (Fig. 4A). The densities corresponding to the terminal SIA and linked galactose became visible at a contour level of 1.1σ, while the density for the subsequent GlcNAc residue was only apparent at lower contour levels (0.8σ or below). No density was observed for the branching fucose or subsequent Gal-(Fuc)-GlcNAc glycan chain; thus, these sugars were not modeled (Fig. 4B). The density for s(Le^x^)_2_ is located within a shallow cavity at the base of the threefold protrusion, surrounded by the twofold depression and 2/5 wall (Fig. 4A). The interactions of sialic acid with the H-1PV capsid are predicted to include a salt bridge between the C1 carboxylate and R381, and multiple hydrogen bonds to R381, R401, and T403, in a similar fashion to LuIII, likely due to the conservation of these residues (Fig. 4D; Table 4). Unlike LuIII, PDBePISA predicted H-1PV’s E400 (LuIII: D393) to be a potential hydrogen bonding partner with the C4 hydroxyl of SIA (Table 4). No interactions of the capsid with GlcNAc were observed. A total of 12 residues are partially or fully buried by the glycan located primarily within a single VP monomer (R381, Y384, D391, K394, E400, R401, Y402, T403, Y564, K569, and D581), with only one contributing residue from a threefold-related chain (N329) (Fig. 5). Overall, the total buried capsid surface area (BSA) by SIA and Gal is calculated to ~180 Å^2^ and 30 Å^2^, respectively.

## DISCUSSION

The ability to bind sialic acid is a common occurrence among parvovirus capsids. To date, 16 capsids of viruses from three genera within the *Parvovirinae* have been identified to bind SIA, *Protoparvovirus*: MVM, CPV, FPV, PPV, H-1PV, LuIII, BuV1, CuV, and TuV (17, 30), *Dependoparvovirus*: AAV1, AAV4, AAV5, AAV6, BAAV, and SAAV (30, 31), and *Bocaparvovirus*: BPV (30). Sialic acids are ubiquitous on vertebrate cell membranes. They are usually found at terminating branches of N-glycans, O-glycans, and glycosphingolipids (32). Specific sialylated glycans such as s(Le^x^) and s(LN) are upregulated in many metastatic cancers and are often associated with poor survival rates (33–35). A number of rodent viruses within the *Protoparvovirus* genus, MVM, LuIII, and H-1PV, display inherent oncolytic activity against some human tumors. Interestingly, all of these viruses are capable of binding to SIA, as shown for LuIII in this study and demonstrated for MVM and H-1PV in previous studies (19, 22). Specifically, the utilization of SIA is shown to occur through recognition of s(Le^x^) or s(LN) motifs by H-1PV and LuIII. This is comparable to MVM, which also prefers larger polysaccharides (36). As a result, LuIII, H-1PV, and MVM may effectively target many of these tumors (2, 13, 37–40).

Previous studies have shown MVM (specifically MVMi, which was not tested here) recognizes polysialylated glycans, such as GD2 (22). In this study, this trait was partially demonstrated for H-1PV and LuIII. However, conflicting results between the two glycan microarrays used in this study suggest that further research is needed to determine if this trait is shared among all three viruses. In addition, while these results identified α2-3-linked SIA as a common receptor for LuIII, H-1PV, and MVM, α2-6-linked SIA was also identified during microarray screening, especially for H-1PV. Discrepancies between the two glycan microarray libraries may arise from differences in the flexible linkers utilized for glycan immobilization on array surfaces. Further validation is needed, as differential recognition of α2-3 and α2-6-linked SIA among rodent protoparvoviruses could have implications in targeting different stages of disease progression, including for glioblastoma or melanoma (35, 41, 42).

LuIII, H-1PV, and MVM utilize adjacent capsid loci for attachment to glycan receptors (2, 16). For LuIII and H-1PV, SIA binds at a common locus near the 3-fold base and 2/5-fold wall, while MVM’s SIA-binding site is located ~9 Å away, deep within the twofold depression (Fig. 5). Residues along the 2/5-fold wall control these differences in SIA-binding locations, as described further below. Although LuIII and H-1PV employ an overlapping capsid region for SIA recognition, the local environments around the contacting residues differ slightly. Further mutagenesis studies are needed to determine the relevance, if any, of these differences in the chemical environment. If the mutation of these adjacent residues is enough to affect binding kinetics, it would therefore contribute to the differences in binding tropism and transduction efficiency between the two viruses, despite their specific contacts with SIA being extremely similar. The nine common residues that were predicted by PDBePISA to become buried upon binding for both LuIII and H-1PV were N323/N329, R375/R381, Y378/Y384, D393/E400, R394/R401, Y395/Y402, T396/T403, R563/K569, V575/D581, respectively (Fig. 5). While most of these residues are conserved or similar in character, V575 in LuIII and D581 in H-1PV, in particular, are not. Located beneath the 2/5-fold wall in LuIII and H-1PV, V575/D581 is closest (~4.6 Å, ~3.4 Å, respectively) to the N-acetyl group of SIA, which is closer to Gal and therefore the most terminal functional group of the entire motif. While not close enough to engage in major interactions, the hydrophobic character of V575 in LuIII could provide a more favorable local environment for the C11 of the N-acetyl group. By contrast, H-1PV’s D581 may have a slightly stronger effect due to both the shorter distance and increased negative charge, a potential contributing factor for H-1PV’s higher binding signals in the Max-Planck glycan array. Since the contact residues shown in Fig. 3 and 4 are conserved, it is possible that these residues located in the periphery contribute to the observed differences in the binding kinetics of SIA. Thus, the varying chemical/environmental conditions adjacent to SIA functional groups, resulting from non-conserved residues between LuIII and H-1PV, will need to be investigated further to determine the extent of their impact on binding efficiency.

The SIA-binding site for MVM has been determined previously and is separate from the one utilized by LuIII and H-1PV (2, 16). Although different residues are involved, all three viruses bind to many of the same α2-3 sialylated glycan motifs (22). This indicates that the ability to bind SIA evolved multiple times among the protoparvoviruses, similar to the AAVs, where at least three different sialic acid-binding sites have been described (31, 43, 44). The different binding characteristics result from residue variability as well as alternate arrangements of VR IX, which flanks the twofold depression. However, one feature that is common among all three viruses is the presence of a ring of polar amino acids responsible for the glycan interaction. Nearby residues also control CPV attachment to SIA, suggesting a common function for this capsid region at the genus level (45). Mutagenesis studies have shown that residues R377, E396, and R397 are required for CPV to bind sialic acid (46). These residues correspond to the contact residues R375/R381, D393/E400, R394/R401 in LuIII/H-1PV, respectively. However, the comparison of the structure of CPV to the glycan-bound LuIII, H-1PV, and MVM structures shows notable differences in the VRs that compose the LuIII/H-1PV and MVM SIA-binding pockets. Taken together, these findings indicate that SIA interactions on the CPV surface occur in a similar region using homologous residues to those found in LuIII and H-1PV. However, it is likely that the glycan chain assumes a different conformation compared to what is found in the LuIII and H-1PV-bound structures due to the structural variations in the surrounding glycan architecture.

### Summary and conclusion

This study provides detailed information on the glycan receptor recognition for two additional members of the *Protoparvovirus* genus. Overall, LuIII’s ability to bind s(Le^x^) and s(LN) explains its tumor tropism, since the virus targets both glioblastoma and melanoma very efficiently (2, 13). Likewise, H-1PV crosses the blood-brain barrier to target glioblastoma, as observed during clinical trials (47). It has been previously shown that the twofold region of MVM and LuIII capsid interacts with specific sialylated glycans, namely α2-3-linked SIA. These glycans are overexpressed on tumors and facilitate their interaction with the virus. There are significant differences in the amino acid sequence and consequently the structure of the twofold depression of MVM, H-1PV, and LuIII. This results in the different binding mode observed between the viruses and their corresponding receptors. To conclude, this work provides a basis for understanding *Protoparvovirus* cell tropism by identifying specific amino acids that control SIA receptor usage among LuIII, H-1PV, and MVM. Shared recognition of Neu5Acα2-3Galβ1-4GlcNAc is conferred mainly by two conserved arginine residues (LuIII/H-1PV 375/381, 394/401), and a conserved threonine residue (396/403), centrally located in the *Protoparvovirus* glycan-binding domain.

## MATERIALS AND METHODS

### Cell culture

All media were supplemented with 10% fetal calf serum and 1% antibiotic-antimycotic (Gibco). *Spodoptera frugiperda* (Sf9) cells were grown at 27°C in suspension in Grace’s medium (Gibco) under constant agitation. Chinese hamster ovary (CHO) cell lines (Pro-5, Lec-2, and Lec-8) were maintained by adherent cell culture (37°C and 5% CO_2_) using α-minimum essential medium (α-MEM) (Gibco).

### Production of recombinant VLPs

The use of the baculovirus expression system has been described for MVM and LuIII VLPs (48). Similarly, the wild-type VP2 sequence for H-1PV, which was previously obtained from the pSR19 clone (19), was transferred to the pFastbac shuttle vector utilizing the *BamH*I and *Xho*I restriction sites. Recombinant baculovirus constructs carrying the VP2 gene were generated according to the manufacturer’s instructions (Bac-to-Bac, Invitrogen, Carlsbad, CA, USA). Site-specific transposition of VP2 into the bacmid expression vector was achieved by transformation of DH10 *Escherichia coli* cells. Initial virus stocks were obtained from plaque purification and amplified by three passages in 50 mL Sf9 suspension cultures, followed by plaque assay to estimate viral titers. These baculovirus stocks were used to infect Sf9 cells (2 × 10^5^ cells/mL) at a multiplicity of infection of 5 in 1 L cultures and harvested 5–7 days post-infection.

### VLP purification by ultracentrifugation

All samples were purified according to published protocols, utilizing an ethanol dry-ice bath for three freeze-thaw cycles, benzonase treatment, 20% sucrose cushion, and a step sucrose gradient ranging from 5% to 40% sucrose (48). In addition, the final step of purification included a cesium chloride gradient for MVM (29). Importantly, buffers used for H-1PV and LuIII purification were supplemented with 500 mM NaCl, 8 mM CaCl_2_, and 2 mM MgCl_2_ to improve protein solubility. To determine sample purity, VLPs were analyzed by 10% sodium dodecyl sulfate polyacrylamide gel electrophoresis (SDS-PAGE), which ran for 15 minutes at 75 V and 1 h at 150 V. The gels were stained with GelCode Blue (ThermoScientific, Waltham, MA, USA). Furthermore, VLP integrity was confirmed using negative stain transmission electron microscopy (EM) (stained with 1% uranyl acetate).

### Fluorescent labeling of VLPs

Samples were prepared by extensive dialysis into 1× phosphate-buffered saline (PBS) supplemented with 500 mM NaCl. This step removed any traces of Tris base, which would inhibit the N-hydroxysuccinimide reaction utilized for capsid labeling. The DyLight488 Labeling Kit (Pierce) was utilized according to the manufacturer’s protocol with some modifications. Upon completion of the 1 h DyLight488 incubation period, each sample received an additional 20 µL of borate buffer and was incubated for 30 additional minutes at room temperature. In place of using the purification resin, samples were dialyzed to remove any excess dye, using 10 mM Tris-HCl, 500 mM NaCl, 8 mM CaCl_2_, and 2 mM MgCl_2_ at pH 7.5. To confirm successful labeling, VLPs were visualized using 10% SDS-PAGE (unstained), followed by imaging under ultraviolet light.

### Glycan microarray screening

LuIII, H-1PV, and MVMp were tested for binding to specific glycan motifs on an array. The chemically defined glycan microarrays were synthesized by immobilization of oligosaccharides onto NHS-activated glass slides, either by the Seeberger laboratory at the Max-Planck Institute or the Consortium for Functional Glycomics (CFG) (49–52). Each glycan is present on the glass slide in replicates of *n* = 3 or *n* = 4, respectively.

In the Seeberger laboratory at the Max-Planck Institute, microarray slides were blocked in 1× PBS supplemented with 30 mM MgCl_2_ and 20 mM KCl (PBS-MK) and 1% bovine serum albumin (BSA) for 1 h at room temperature. The slides were washed three times with PBS-MK and dried by centrifugation (5 min; 300 × *g*). DyLight488-labeled VLPs for LuIII, H-1PV, and MVMp were added to slides and incubated overnight (4°C) in 0.01% Tween 20-PBS MK in a humid chamber. Afterward, the microarray slides were washed with 0.1% Tween 20-PBS-MK three times, rinsed in water, and dried by centrifugation. Fluorescent signal excited at 488 nm (indicating VLP binding) was detected using a GenePix 4300A microarray scanner (Molecular Devices), and signal intensities were determined by GenePix Pro 7 software (Molecular Devices). The photomultiplier tube (PMT) voltage was adjusted so that scans were free of saturation signals, and mean fluorescence intensity (MFI) values were exported to Microsoft Excel. DyLight488-labeled VLPs were submitted for screening on the same microarray, using concentrations of 0.1 mg/mL (LuIII and H-1PV) or 0.4 mg/mL (MVM).

At the CFG, unlabeled VLPs and empty virus particles were submitted for glycan microarray screening. LuIII VLPs were screened at a concentration of 0.2 mg/mL on the Mammalian Printed Array Version 4.1. H-1PV particles were purified as previously reported (29) and assayed at a concentration of 0.4 mg/mL using the Mammalian Printed Array Version 3.1 (array availability differed based on the time of sample submission). Microarray slides were washed and blocked using 50 mM ethanolamine in 0.1 M Tris-HCl (pH 9.0), followed by rehydration in 20 mM Tris-HCl, 150 mM NaCl, 0.2 mM CaCl_2_, and 0.2 mM MgCl_2_ (TSM) for 5 minutes. The unlabeled samples were diluted in 1× PBS and incubated on microarray slides for 1 h at room temperature, washed in 0.05% Tween 20-TSM, and incubated with polyclonal rabbit serum directed against the viral capsid (1:5000 dilution) for 1 h. Slides were rinsed four times in 0.05% Tween 20-TSM, followed by four additional washes in TSM without Tween. Alexa-488-labeled anti-rabbit IgG antibody was added to slide surfaces for 1 h at room temperature, and array slides were washed again prior to signal detection by a Perkin Elmer ProScanarray microarray scanner. The image analysis software, ScanArray Express, was used to determine average relative fluorescence units (RFU) and standard deviation values.

### Cellbinding assay

Chinese hamster ovary (CHO) cell lines were grown to 70%–90% confluency, 5 mM EDTA, and washed with serum-free α-MEM media. On the same day, resuspended cells were diluted to 5 × 10^5^ cells per mL and pipetted into 1.5 mL centrifuge tubes. The cells were chilled on ice for a minimum of 15 mins preceding the addition of DyLight488-labeled LuIII, H-1PV, or MVMp VLPs at a ratio of 1 × 10^5^ per cell. Cell binding was completed at 4°C for 30 min, with continuous mixing. Afterward, the pelleted cells were collected by centrifugation at 3,000 rpm for 10 mins in an F45-24-11 rotor (Eppendorf), washed twice in chilled 1× PBS, pelleted again, and resuspended in serum-free media for detection of fluorescence at 488 nm by flow cytometry on the BD FACSCalibur. Each condition was tested in duplicate during three separate experiments.

### Sample preparation for Cryo-EM

VLPs were concentrated by pelleting at 50,000 rpm on a SW55Ti rotor (Beckman Coulter, Brea, CA, USA) for 1 h at 4°C, and resuspended in buffer containing 10 mM Tris-HCl, 500 mM NaCl, 8 mM CaCl_2_, 2 mM MgCl_2_, at pH 7.5. This resulted in final VLP concentrations of 0.8 mg/mL (for LuIII) and 0.9 mg/mL (H-1PV). Synthesized glycans were provided by Core D of the CFG for complexing with VLPs. Previously, s(LN)_3_ was reconstituted in deionized water (leading to a final pH of ~2.0 prior to mixing with VLPs). The glycans were added to VLPs in solution at a molar ratio of 1:20 (VP to glycan) for LuIII or 1:100 for H-1PV and incubated at 4°C for a minimum of 1 h prior to cryo-preservation. The VLP-glycan complexes were pipetted (3 µL aliquots) onto glow-discharged copper grids containing carbon support over the holes (Quantifoil R 2/2 400 mesh, Electron Microscopy Sciences). All samples were vitrified using a Vitrobot Mark 4 (FEI) operated at 95% humidity and 4°C. The cryo-preserved grids were screened on a Tecnai G2 F20-TWIN microscope operated under low-dose conditions (200 kV, ~20 e^−^/Å^2^), with images collected on a Gatan UltraScan 4000 CCD camera (Gatan, Inc., Pleasanton, CA, USA).

### Cryo-EM data collection

The data sets were collected using Leginon (ref) on a Titan Krios electron microscope (FEI) equipped with a Gatan post-column imaging filter (GIF) at a slit width of 20 eV. The microscope was operated at 300 kV, using 130,000× magnification and a pixel size of 1.1 Å. Movies were recorded into movie stacks of 50 frames per micrograph at a nominal total dose of 75 e^-^ per A^2^. The Gatan K2 Summit direct electron detection camera was operated under counting mode. All data were collected as part of the NIH “West/Midwest Consortium for High-Resolution Cryo Electron Microscopy” project.

### Structure determination

The program cisTEM was utilized for 3D reconstruction of the LuIII and H-1PV data sets. Initially, the movie frames were aligned, imported, and their contrast transfer function (CTF) parameters estimated. The CTF output was used to sort the micrographs based on quality (48, 53–60). Automatic particle picking, using a particle radius of 130 Å, produced a set of particles that were subjected to 2D classification, eliminating non-viral debris from the picking process. Following 2D classification, cryo-EM maps of the VLPs were reconstructed, beginning with ab initio 3D model generation. This was followed by auto refinement and density map sharpening using a pre-cutoff B-factor value of −90 Å², and variable post-cutoff B-factor values including 0, 20, 50, and 150 Å². The resolutions of the cryo-reconstructed maps were estimated based on a Fourier Shell Correlation (FSC) of 0.143. The maps were then resized using the EMAN2 (61) subroutine e2proc3d.py based on best-fit parameters determined by correlation coefficients from Chimera. After conversion to the CCP4 format using MAPMAN (62), models of the 60mers generated using ViperDB (63) were fitted into the density maps using Chimera and refined using the −90 Å²/0 Å² sharpened maps. Amino acid main chains and side chains were manually assigned via the real-space refinement tool in Coot and automatically refined in Phenix, which also provided the final refinement statistics. Finally, 60mers of each model were generated using ViperDB (64) and docked into the cryo-EM density map with Chimera using the “Fit in Map” option to confirm the Phenix output.

### Identification and comparison of receptor binding sites

Atomic models generated for each VLP-glycan complex were visualized in Coot to identify capsid loci of interest (56). Individual protein chains were extracted from the icosahedral 60mer for analysis by the online tool PDBePISA (http://www.ebi.ac.uk/pdbe/pisa/) to identify capsid residues located within the interface between VP and SIA (65). These VP2 positions were further examined in Coot by SSM superposition of all four structures, enabling residue-by-residue comparison (56). The fit-in-map function in Chimera was also utilized to align the cryo-EM maps obtained from different data sets (57).

## Data Availability

All reconstructed maps and atomic models have been deposited in the Electron Microscopy Data Bank (EMDB) and the PDB, respectively, using the following accession numbers: LuIII-s(LN)_3_ (9NAJ and EMD-49196), LuIII-s(Le^x^)_2_ (9NAI and EMD-49195), H-1PV-s(Le^x^)_2_ (9NBG and EMD-49228), and H-1PV (9NAW and EMD-49205).

## SUPPLEMENTAL MATERIAL

Fig. S1 jvi.00297-25-s0001.docx Comparison of the H-1PV crystallographic and cryo-EM structures.

ASM does not own the copyrights to Supplemental Material that may be linked to, or accessed through, an article. The authors have granted ASM a non-exclusive, world-wide license to publish the Supplemental Material files. Please contact the corresponding author directly for reuse.
