# Supplementary material for: Mapping the sialic acid-binding sites of LuIII and H-1 parvovirus
Source: J Virol. 2025 Jul 24;99(8):e00297-25. doi: 10.1128/jvi.00297-25 (PMC12363179; doi:10.1128/jvi.00297-25)
Supplement: Fig. S1 — Comparison of the H-1PV crystallographic and cryo-EM structures. [file jvi.00297-25-s0001.docx]

**
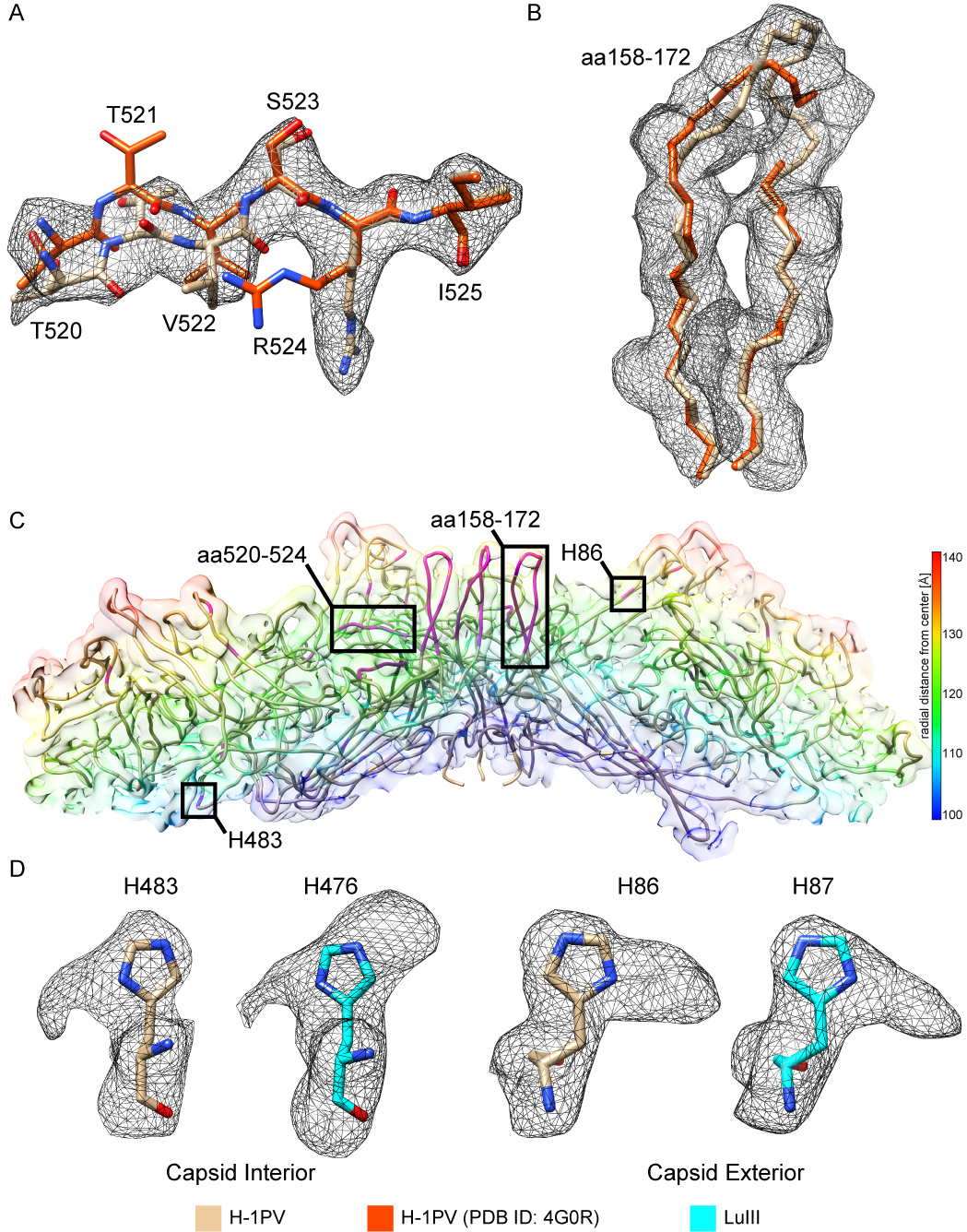
**

**Supplemental Figure S1.** Comparison of the H-1PV crystallographic (PDB ID: 4G0R) and cryo-EM (PDB ID: 9NAW) structures. **(A)** SSM superposition visualizing side chain differences within the HI loop. **(B)** SSM superposition focusing on the Cα backbone within the DE loop. **(C)** The central section of the reconstructed map for 9NAW is shown in surface representation (colored by increasing distance from the capsid interior) with the VP2 60mer shown in tan (cartoon representation). All visible HI and DE loops, as well as histidine residues displaying extra density, are shown in magenta. **(D)** Comparison of homologous histidine residues containing extra density within 9NAW and 9NAJ (LuIII-s(LN)_2_). 4G0R is shown in orange, 9NAW (H-1PV) in tan, and 9NAJ (LuIII) in cyan. Images were generated using Chimera.
